# 3,5 Diiodo-L-Thyronine (T2) Does Not Prevent Hepatic Steatosis or Insulin Resistance in Fat-Fed Sprague Dawley Rats

**DOI:** 10.1371/journal.pone.0140837

**Published:** 2015-10-20

**Authors:** Daniel F. Vatner, Jaclyn Snikeris, Violeta Popov, Rachel J. Perry, Yasmeen Rahimi, Varman T. Samuel

**Affiliations:** 1 Department of Internal Medicine,Yale University School of Medicine, New Haven, CT, United States of America; 2 Department of Cellular & Molecular Physiology, Yale University School of Medicine, New Haven, CT, United States of America; 3 West Haven VAMC, West Haven, CT, United States of America; INSERM/UMR 1048, FRANCE

## Abstract

Thyroid hormone mimetics are alluring potential therapies for diseases like dyslipidemia, nonalcoholic fatty liver disease (NAFLD), and insulin resistance. Though diiodothyronines are thought inactive, pharmacologic treatment with 3,5- Diiodo-L-Thyronine (T2) reportedly reduces hepatic lipid content and improves glucose tolerance in fat-fed male rats. To test this, male Sprague Dawley rats fed a safflower-oil based high-fat diet were treated with T2 (0.25 mg/kg-d) or vehicle. Neither 10 nor 30 days of T2 treatment had an effect on weight, adiposity, plasma fatty acids, or hepatic steatosis. Insulin action was quantified *in vivo* by a hyperinsulinemic-euglycemic clamp. T2 did not alter fasting plasma glucose or insulin concentration. Basal endogenous glucose production (EGP) rate was unchanged. During the clamp, there was no difference in insulin stimulated whole body glucose disposal. Insulin suppressed EGP by 60% ± 10 in T2-treated rats as compared with 47% ± 4 suppression in the vehicle group (p = 0.32). This was associated with an improvement in hepatic insulin signaling; insulin stimulated Akt phosphorylation was ~2.5 fold greater in the T2-treated group as compared with the vehicle-treated group (p = 0.003). There was no change in expression of genes thought to mediate the effect of T2 on hepatic metabolism, including genes that regulate hepatic lipid oxidation (*ppara*, *carnitine palmitoyltransferase 1a*), genes that regulate hepatic fatty acid synthesis (*srebp1c*, *acetyl coa carboxylase*, *fatty acid synthase*), and genes involved in glycolysis and gluconeogenesis (*L-pyruvate kinase*, *glucose 6 phosphatase*). Therefore, in contrast with previous reports, in Sprague Dawley rats fed an unsaturated fat diet, T2 administration failed to improve NAFLD or whole body insulin sensitivity. Though there was a modest improvement in hepatic insulin signaling, this was not associated with significant differences in hepatic insulin action. Further study will be necessary before diiodothyronines can be considered an effective treatment for NAFLD and dyslipidemia.

## Introduction

Over the past decade, the burden of nonalcoholic fatty liver disease (NAFLD) has been rising. NAFLD is the most common chronic liver disease and is associated with a spectrum of illness from insulin resistance to type 2 diabetes (T2D) and steatohepatits to cirrhosis and hepatocellular carcinoma. NAFLD is projected to overtake hepatitis C as the leading cause of liver transplantation [1, 2]. Novel pharmaceutical agents are needed to treat NAFLD. Thyroid hormone analogues may be one such therapeutic class. Thyroid hormone can decrease hepatic lipid content by multiple mechanisms, including induction of hepatic CPT-1α leading to an increase in hepatic lipid oxidation [3], and increasing energy expenditure through futile substrate cycling [4–6] and excess thermogenesis [7]. The hepatic actions of thyroid hormone have the additional benefit of lowering plasma lipids. Proposed mechanisms for the action of thyroid hormone on the plasma lipid profile include increased LDL receptor expression [8, 9], decreased SREBP-1c expression [10, 11], increased SREBP-2 expression [12], LDL receptor independent reduction in apoB [13], and increased reverse cholesterol transport, with increased Cyp7A1 activity in rodent models [8, 10]. The use of thyroid hormone (T4 or T3) *per se* is precluded by toxicities including tachyarrhythmias, pulmonary hypertension, osteoporosis, agitation, and even psychosis. Liver-specific and thyroid hormone receptor β (TRβ) -selective agonists [14, 15] have been developed over the past 10–15 years with promising pre-clinical and clinical data. However, unanticipated adverse effects have prevented several of these TRβ selective agents from progressing to clinical use. We have demonstrated that while TRβ agonists effectively decrease hepatic steatosis, they drive increased insulin resistance by multiple mechanisms [16].

A naturally occurring product of thyroid hormone metabolism, 3,5-diiodo-L-thyronine (T2), has been proposed as an alternative thyromimetic. Unlike T3 and T4, T2 does not bind to thyroid hormone receptors; by most measures *in vivo*, in cell culture, and in purified preparations of protein and DNA, T2 has at least 100-fold lower affinity for nuclear thyroid hormone receptors as compared with the active triiodothyronine (T3) [17]. T2 has been shown to improve oral glucose tolerance in fat-fed rats when administered at low pharmacologic doses (250 μg/kg-d) [18]. Furthermore, T2 treatment has been reported to reduce hepatic steatosis in a fat-fed rat model by uncoupling mitochondria and by altering expression of genes encoding enzymes involved in hepatic lipid oxidation, lipid storage, and lipid export [19, 20]. The lipid-depleting potential of T2 has been replicated in primary hepatocytes [21]. Though T2 has been proposed as a treatment for dyslipidemia, the dose of T2 required for meaningful lipid lowering also worsens angina in patients with coronary artery disease [22]. In rodents, a similar dose induces cardiomegaly and suppresses TSH [13]. These physiological effects are consistent with TRβ activation and reminiscent of the toxicities associated with thyroid hormone excess [23]. There are some reports that lower doses (250 μg/kg-d) can improve glucose tolerance, but a broader preclinical evaluation of these lower doses remain to be reported.

In the present study, we evaluated the effects of low-dose T2 treatment (250 μg/kg-d) in high fat (safflower oil) fed, male Sprague-Dawley rats. Insulin sensitivity was quantified using the hyperinsulinemic-euglycemic clamp in awake, unrestrained rats. These studies allowed us to investigate the mechanisms by which T2 may regulate hepatic lipid metabolism and quantify the effects of T2 on tissue-specific insulin sensitivity.

## Materials and Methods

### Animals

Male Sprague-Dawley rats (250–275g), were obtained from Harlan Laboratories and acclimated for at least 3 days and randomly assigned to treatment groups before studies commenced. Rats were housed in accordance with the Guide for the care and use of laboratory animals and standard operating procedures of the Yale Animal Resource Center. Specifically, they were multiply housed (2-3/standard cage, when possible) at 72±2°F, 30–70% humidity, with a 12:12 hr light/dark cycle, in individually vented cage racks on corncob bedding. Pine shavings, gnawing blocks, Solo cups, Enviro-dri (shredded paper) and Fat Rat Huts are provided for enrichment. Chlorinated water is provided either by automatic water or bottles. After rats underwent the placement of jugular venous (for blood sampling) and carotid artery (for infusion) catheters, they were singly housed for 3–5 days before the initiation of T2 treatment. Rats were placed on a 27% (w/w) safflower oil high-fat diet (Dyets 112245: 26% carbohydrate, 59% fat, 15% protein calories, Dyets, Inc., Bethlehem, PA) during the treatment period. For T2 treated animals, 3,5-diiodo-L-thyronine (Sigma, St. Louis, MO) was dissolved in 10 mM sodium hydroxide and neutralized in phosphate buffered saline (to render it soluble), and was injected intraperitoneally at a dose of 250 μg-kg^-1^-d^-1^ for ten days. Vehicle treated animals were treated identically to T2 treated animals, except T2 was not included in the intraperitoneal injections. Injections were given between 0900–1200H (light cycle) to animals in their home cage. Body weight was monitored three to four times weekly. Experiments were performed 16–24 hours after the final injection of T2.

All procedures were approved by the Institutional Animal Care and Use Committee of the Yale University School of Medicine. This study was carried out in strict accordance with the recommendations in the Guide for the Care and Use of Laboratory Animals of the National Institutes of Health. Surgeries were performed under isoflurane anesthesia, and carprofen analgesia was provided in the postoperative period. All euthanasia was performed either with intravenous pentobarbital, or under isoflurane anesthesia. Care was taken throughout the study to minimize suffering.

Group sizes of 7–12 rats were used for each arm of each experiment. Specifically, 22 rats were studied in the ten day basal animal experiment (10 vehicle-treated vs. 12 T2-treated); 16 rats were studied in the four week basal animal experiment (8 vehicle vs. 8 T2); and 15 rats were studied by hyperinsulinemic-euglycemic clamp (7 vehicle vs. 8 T2).

### Tissue triglyceride isolation and measurement

Triglycerides were extracted using 100–200 mg tissue. Tissues were homogenized in ice cold 2:1 chloroform:methanol, and lipids were extracted with shaking at room temperature for 3–4 hours. H_2_SO_4_ was added to ~100 mM, samples were vortexed then centrifuged to achieve phase separation. The organic phase was collected, dried down, and resuspended in chloroform. TG content was measured using the Sekisui Triglyceride-SL kit (Sekisui Diagnostics, Lexington, MA).

### Hyperinsulinemic-euglycemic clamp studies

For all assessments groups of T2 and vehicle treated rats were assessed at the same time. Clamp studies in rats were performed after an overnight fast as previously described [16, 24]. The basal period began with a prime (2.0 mg / kg over 3 min) of 99% labeled [6,6-^2^H]glucose, followed by a continuous infusion at a rate of 0.2 mg / kg per minute for 2 hr to assess basal glucose turnover. After the basal period, the hyperinsulinemic-euglycemic clamping was conducted for 120 min with a primed/continuous infusion of human insulin (40 mU / (kg-min) for 3 min) / [4 mU / (kg-min)] (Novo Nordisk Inc., Denmark) and a variable infusion of ~20% dextrose to maintain euglycemia (approximately 110 mg/dl). The 20% dextrose was enriched with [6,6-^2^H]glucose to approximately 2.5% to match the enrichment in the plasma achieved after the basal period (i.e. “hot-GINF”). At the end of the clamp, rats were anesthetized with sodium pentobarbital injection (75 mg / kg), and all tissues were taken within 3 min, frozen immediately using cooled aluminum tongs in liquid N_2_, and stored at -80°C for the subsequent analysis.

### Western blotting

Tissue (~100 mg) was homogenized in 1 ml ice-cold homogenization buffer (20 mM Tris-HCl, pH 7.4, 5 mM EDTA, 0.25 mM EGTA, 10 mM Na4P2O7, 1% NP-40, protease and phosphatase inhibitor cocktails (Roche Diagnostics, Indianapolis, IN)), and centrifuged at 6,000 rcf at 4°C for 15 minutes. The supernatant was taken, and protein concentration was determined by the Bradford-coomassie method (Thermo Scientific, Waltham, MA). 100 μg of protein were loaded and resolved by SDS-PAGE using a 4–12% gradient gel or a 15% gel and electroblotted onto polyvinylidene difluoride membrane (DuPont, Boston, MA) using a semi-dry or wet-transfer cell. The membrane was then blocked for 60 minutes at room temperature in wash buffer containing 5% (w/v) nonfat dried milk or bovine serum albumin, and incubated overnight with primary antibody. After washing, membranes were incubated with horseradish peroxidase-conjugated secondary antibody (Cell Signaling Technology, Danvers, MA) for 60 minutes. Detection was performed with enhanced chemiluminescence.

For PKCε translocation, cytoplasm and plasma membrane were separated by ultracentrifugation as previously described [25, 26], and Western blotting was performed as described above.

Akt, phosphorylated Akt (Ser473), PKCε, phosphorylated Jnk, and BiP antibodies were purchased from Cell Signaling Technology (Danvers, MA). Sodium potassium ATPase antibody and Calnexin antibody were purchased from Abcam Inc. (Cambridge, MA). GAPDH and Caspase 1 p10 antibodies were purchased from Santa Cruz Biotechnology Inc. (Dallas, TX). Jnk antibody was purchased from EMD/Millipore/Upstate. β-actin antibody was purchased from Sigma (St. Louis, MO).

### Quantitative PCR

Total RNA was extracted from ~15 mg liver using RNeasy mini kit (Qiagen, Valencia, CA). RNA was reverse-transcribed into cDNA with the use of the QuantiTect Reverse Transcription Kit (Qiagen, Valencia, CA). The abundance of transcripts was assessed by real-time PCR on an Applied Biosystems 7500 Fast Real-Time PCR System (Applied Biosystems, Carlsbad, CA) with a SYBR Green detection system (Stratagene, La Jolla, CA). The expression data for each gene of interest were against β-actin as the invariant control and relative expression determined using amplification efficiencies [27]. Primer sequences are shown in Table 1.

**Table 1:** qPCR primer sequences.

Gene	Forward	Reverse
β-actin	CCAGATCATGTTTGAGACCTTC	CATGAGGTAGTCTGTCAGGTCC
ACC	AGCAGATCCGCACTTG	ACCTCTGCTCGCTGAGTGC
Adiponectin	GCAACCGAAGGGCCAGGAGC	TCTGCCATCACGGCCCGGTA
CPT1a	GGGAGTGCAGAGCAATAGGTCCC	ACCTCTGCTCGCTGAGTGC
Deiodinase 1	TGGTTCGTCCTGAAGGTCCGC	TCAACACCAGGGGTCTGCTGC
DGAT	AGGCCTTGATGGTTTCTATCCA	GCTGCCCTTCCCCAATTAAC
Fatty Acid Synthase	CCAGGAACTGAACGGCATTAC	GATTTGGTGGAGCCAATTAACAA
FGF21	TGCTTAAGGACGGATACAATGTGT	CTGGATCCTGGGAGTCCTTCT
G6Pase	GAAGGCCAAGAGATGGTGTGA	TGCAGCTCTTGCGGTACATG
Hairless	GCCCAGCGTATCCGTCGCTTT	AACCTGCGTCGCAACCCTGA
Leptin	TGGACCTTAGCCCTGAATGCT	CGTGCGGATAACTTTAGTCAAGCC
NCoR1	AGTCGCTACAGCCCAGAGTC	CTCCTCTCTGGGGATTTTCC
Nrip1	CCACAGTCAAGCAAACTGGC	AGGAACACCGCACATTGGAT
PPARα	CAGGAGAGCAGGGATTTGCA	CCTAGGCTCAGCCCTCTTCA
PGC1-α	ATGAGAAGCGGGAGTCTGAA	GCGGTCTCTCAGTTCTGTCC
SREBP-1c	CGCCCATGAGTCGAGTAAGC	CGTCCCAGACATACACCAA
Trb3	CTTTTGGAACGAGAGCAAGG	GGGTCTTCGTGAAAAAGGTG

ACC: Acetyl CoA Carboxylase; CPT1a: Carnitine Palmitoyltransferase-1A; DGAT2: Diacylglycerol O-acyltransferase 2; FGF21: Fibroblast Growth Factor 21; G6Pase: Glucose 6 Phosphatase; NCoR1: Nuclear receptor corepressor 1; Nrip1: Nuclear receptor interacting protein 1; PPARα: Peroxisome proliferator-activated receptor alpha; PGC1-α: Peroxisome proliferator-activated receptor gamma coactivator 1-alpha; SREBP-1c: Sterol regulatory element binding protein 1c; Trb3: Tribbles pseudokinase 3.

### Biochemical analysis

Plasma glucose concentrations measured during clamp studies were measured using a YSI 2700 (YSI Life Sciences, Yellow Springs, OH). Plasma insulin concentrations were determined by radioimmunoassay using the LINCOplex Assay system (Millipore). Non-esterified fatty acids were using the NEFA-HR Color A and B reagent test kit (Wako Chemicals USA, Inc., Richmond, VA).

### Statistical Analysis

For most comparisons, the average value for each specific parameter inT2 treated animals was compared to vehicle treated animals. Animals were either in the basal (fasting) state or at the end of the hyperinsulinemic-euglycemic clamp. For some analysis, the change between clamped and basal state (e.g. increase in insulin receptor phosphorylation) was assessed for each group. Statistical analysis of the data was performed using GraphPad Prism 5.0.3. Data were compared using Student’s unpaired T-test. All data are expressed as mean ± SE unless otherwise indicated. P values less than 0.05 were considered significant.

## Results

### T2 administration had no effect on fasting (basal) parameters

Male Sprague Dawley rats were fed a high fat diet for ten days, with concomitant IP administration of 250 μg/kg T2 or vehicle daily. In contrast to prior reports [18–20], T2 had no effect on body weight gain, plasma nonesterified fatty acid concentration (NEFA), or accumulation of hepatic triglyceride (Table 2). There was no effect on the weight of the epididymal white adipose tissue pad (Table 2). Neither fasting glucose nor insulin concentration was affected (Table 2). As most prior reports of pharmacologic use of T2 used a one month treatment period, these experiments were repeated in male Sprague Dawley rats fed a safflower oil high fat diet for one month, with concomitant IP administration of 250 μg/kg T2 or vehicle daily. However, even this longer treatment schedule did not change basal parameters (Table 3).

**Table 2:** Overnight fasting parameters, ten days T2 treatment.

	Vehicle treated	T2 treated	*p*
Weight (g)	295 ± 6	297 ± 4	0.78
Hepatic Triglyceride (mg/g liver)	20 ± 2	21 ± 2	0.82
WAT Weight (g)	2.8 ± 0.2	2.6 ± 0.2	0.47
NEFA (mM)	0.44 ± 0.02	0.41 ± 0.03	0.42
Glucose (mg/dL)	105 ± 2	110 ± 2	0.16
Insulin (μU/mL)	12 ± 3	13 ± 2	0.70

Values are means ± SE. *n* = 7–17 per group.

**Table 3:** Six-hour fasting parameters, four weeks T2 treatment.

	Vehicle treated	T2 treated	*p*
Weight (g)	455 ± 16	469 ± 14	0.52
Hepatic Triglyceride (mg/g liver)	37 ± 4	46 ± 5	0.13
WAT Weight (g)	7.5 ± 0.8	8.0 ± 0.8	0.67
NEFA (mM)	0.31 ± 0.04	0.32 ± 0.03	0.87
Glucose (mg/dL)	159 ± 9	160 ± 8	0.90
Insulin (μU/mL)	30 ± 4	32 ± 3	0.76

Values are means ± SE. *n* = 6–8 per group.

### T2 administration had minimal effect on insulin sensitivity

**Fig 1:**
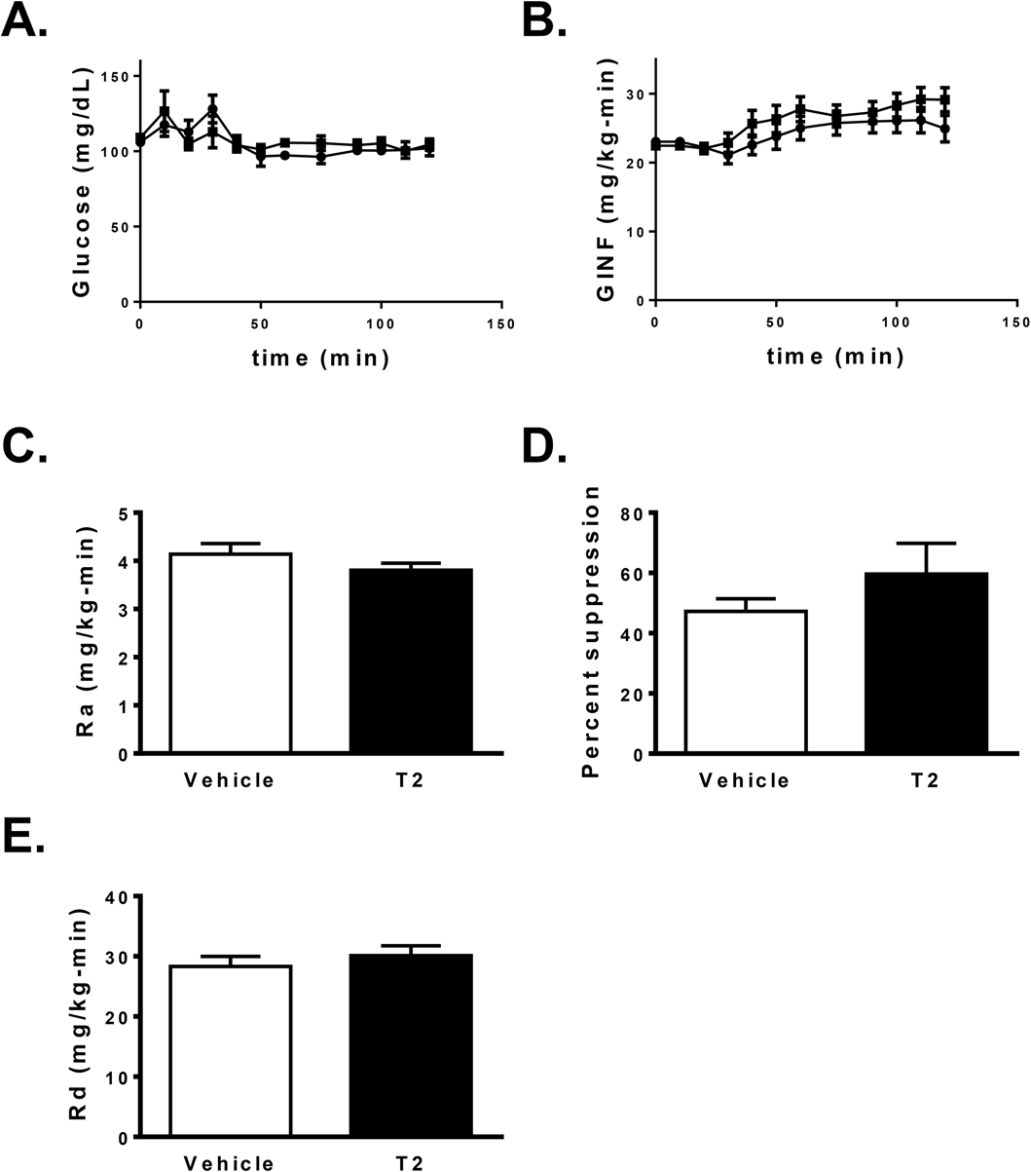
Assessment of tissue specific insulin sensitivity by hyperinsulinemic-euglycemic clamp, comparison between ten days T2 treatment (*n* = 10) vs. vehicle (*n* = 8). A. Plasma glucose and B. glucose infusion rate during hyperinsulinemic-euglycemic clamp (circles = vehicle treatment; squares = T2 treatment). C. Endogenous glucose production under basal conditions (white bars = vehicle; black bars = T2). D. Percent suppression of endogenous glucose production by insulin (4 mU/kg-min). E. Whole body insulin-stimulated glucose disposal.

To assess changes in hepatic and peripheral insulin sensitivity after T2 treatment, hyperinsulinemic-euglycemic clamp studies were performed after 10 days of safflower oil high fat feeding and 250 μg/kg-d T2 treatment in awake, unrestrained rats. Under basal (fasting) conditions, there was no difference in endogenous glucose production (EGP) with T2 treatment (vehicle: 4.14 ± 0.22 mg/kg-min; T2: 3.80 ± 0.15; *P* = 0.21; Fig 1C). There was a 10% increase in the glucose infusion rate required to maintain euglycemia (Fig 1A and 1B) in T2 treated rats (vehicle: 25.8 ± 0.9 mg/kg-min; T2: 28.5 ± 0.9 mg/kg-min; *P* = 0.03), but only a trend towards an improvement in hepatic insulin sensitivity, as assessed by suppression of EGP by insulin (vehicle: 47.3 ± 4.2% suppression; T2: 59.6 ± 10.2% *P* = 0.32; Fig 1D). Peripheral insulin sensitivity, as assessed by whole body insulin stimulated glucose disposal, was unchanged with T2 treatment (*P* = 0.46; Fig 1E).

### T2 treatment affects hepatic insulin signaling through the Akt2 pathway without altering PKCε translocation

**Fig 2:**
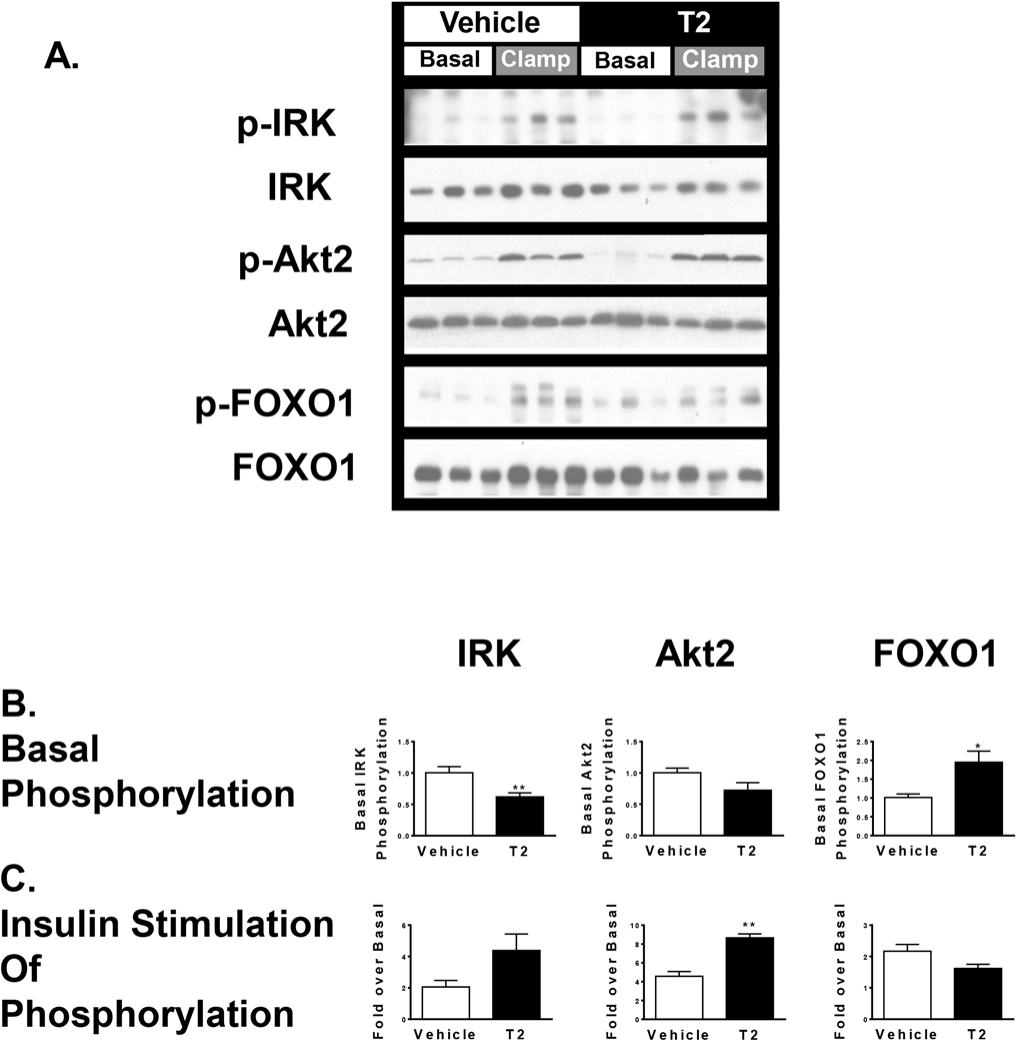
Hepatic insulin signaling through insulin receptor kinase phosphorylation, Akt2 phosphorylation, and FOXO1 phosphorylaiton, assessed by Western blot. A. Representative immunoblots. B. Fasting (unstimulated) phosphoprotein abundance normalized to total protein abundance. C. Protein phosphorylation in the insulin stimulated state relative to the basal (fasting) state. (* P < 0.05; ** p < 0.01 T2 treatment vs. vehicle treatment; *n* = 4–6 per group).

We assessed insulin signaling by quantifying insulin-stimulated changes in phosphorylation of three key components of the insulin signaling pathways: the insulin receptor kinase (IRK), Akt2, and FOXO1 (Fig 2). While phosphorylation of these proteins in fasted rats was low, T2 treatment reduced basal (fasting) IRK phosphorylation by 40% (*P* < 0.01) while increasing FOXO1 phosphorylation by 2-fold (*P* < 0.05); there was a trend toward a 30% reduction in Akt2 phosphorylation (*P* = 0.1). Tissues obtained at the end of the hyperinsulinemic-euglycemic clamp were used to assess insulin stimulated phosphorylation of these proteins. T2 treatment resulted in a trend towards a 2-fold increase in insulin stimulated IRK phosphorylation (*P* = 0.07) relative to the average basal IRK phosphorylation, an increase in Akt2 phosphorylation over basal by 160% (*P* < 0.01), and no increase in phosphorylation of FOXO1.

**Fig 3:**
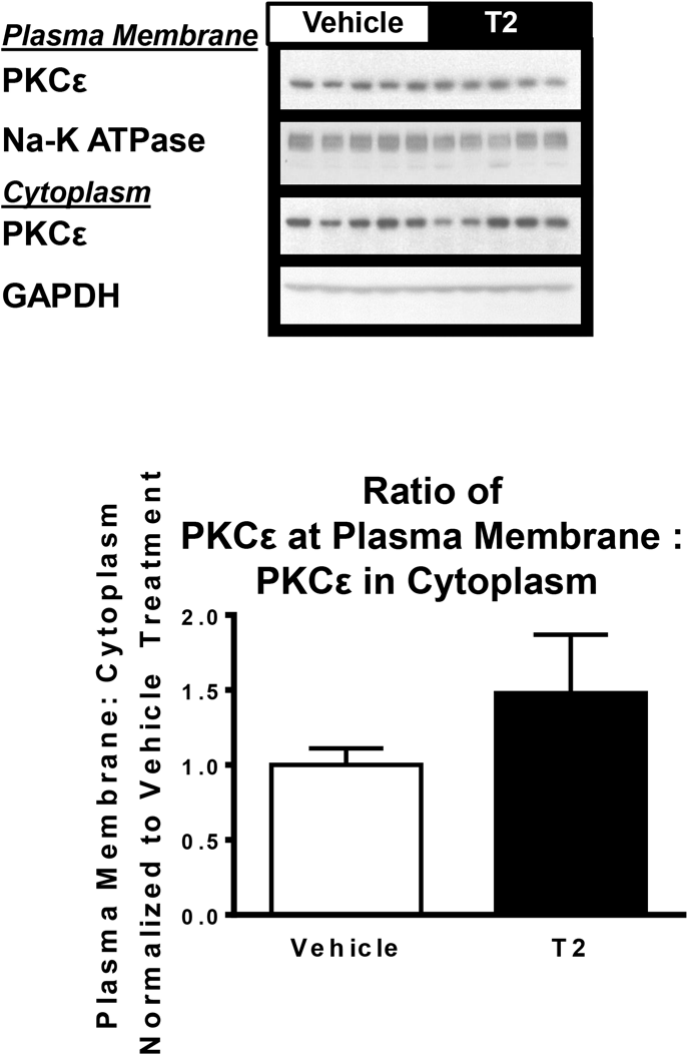
Hepatic PKCε translocation between cytoplasm and plasma membrane. Assessed by Western blot. Plasma membrane PKCε abundance normalized to sodium potassium ATPase, cytoplasmic PKCε abundance normalized to glyceraldehyde 3 phosphate dehydrogenase (GAPDH). Ratio between plasma membrane and cytoplasm PKCε was determined for all samples, this ratio for all samples was normalized to the average ratio in the vehicle treated group. (*n* = 5 per group).

In many models of hepatic steatosis, diacylglycerol mediated activation of PKC impairs hepatic insulin signaling [28]. PKCε membrane translocation, an index of PKCε activation, was unchanged in T2 treated animals (*P* = 0.27; Fig 3), consistent with the lack of change in liver triglyceride content and lack of change in ability of insulin to suppress endogenous glucose production.

### T2 treatment and gene expression

Nuclear thyroid hormone receptor (TR) activation was assessed by quantifying the expression of key TR-responsive genes: *deiodinase 1* in liver [29] and *hairless* in white adipose tissue [16, 30]. There was no detectable increase in the expression of these TR target genes (Table 4), supporting the conclusion that T2 treatment in this dose range does not activate TR.

**Table 4:** qPCR table.

Relative expression	Vehicle treated	T2 treated	*p*
Dio1	1.0 ± 0.04	1.1 ± 0.17	0.62
Hrl (WAT)	1.0 ± 0.23	0.81 ± 0.22	0.58
SREBP-1c	1.0 ± 0.17	1.1 ± 0.11	0.65
ACC	1.0 ± 0.14	1.4 ± 0.26	0.25
FAS	1.0 ± 0.19	0.69 ± 0.05	0.14
DGAT2	1.0 ± 0.17	1.4 ± 0.18	0.16
G6Pase	1.0 ± 0.17	1.5 ± 0.28	0.18
L-PK	1.0 ± 0.38	0.91 ±0.16	0.82
PPARα	1.0 ± 0.27	1.2 ± 0.30	0.62
PGC1-α	1.0 ± 0.12	0.88 ± 0.15	0.56
CPT1a	1.0 ± 0.14	0.79 ± 0.08	0.23
FGF21 (liver)	1.0 ± 0.22	0.53 ± 0.10	0.06
FGF21 (WAT)	1.0 ± 0.17	1.5 ± 0.35	0.24
Adiponectin	1.0 ± 0.14	1.2 ± 0.24	0.42
Leptin	1.0 ± 0.43	0.57 ± 0.17	0.43
NCoR1	1.0 ± 0.13	1.1 ± 0.18	0.73
Nrip1	1.0 ± 0.16	0.83 ± 0.08	0.34
Trb3	1.0 ± 0.18	0.86 ± 0.19	0.60

Dio1: Deiodinase 1; Hrl: Hairless; WAT: White adipose tissue; SREBP-1c: Sterol regulatory element binding protein 1c; ACC: Acetyl CoA Carboxylase; FAS: Fatty acid synthase; DGAT2: Diacylglycerol O-acyltransferase 2; G6Pase: Glucose 6 Phosphatase; L-PK: L-Pyruvate kinase; PPARα: Peroxisome proliferator-activated receptor alpha; PGC1-α: Peroxisome proliferator-activated receptor gamma coactivator 1-alpha; CPT1a: Carnitine Palmitoyltransferase-1A; FGF21: Fibroblast growth factor 21; NCoR1: Nuclear receptor corepressor 1; Nrip1: Nuclear receptor interacting protein 1; Trb3: Tribbles pseudokinase 3. *n* = 4–10 per group.

T2 putatively activates the SIRT1, leading to deacetylation and activation of PGC-1α and SREBP-1c which then alter expression of genes involved in of glucose metabolism and fat metabolism [18]. Thus, we measured the expression of genes thought to be important for T2 action (Table 4). Expression of the glycolytic gene *L-PK* and the gluconeogenic gene *glucose 6-phosphatase* were unchanged. There was a non-significant tendency towards decreased expression of *fatty acid synthase* (*P* = 0.14), but *acetyl coa carboxylase* expression was unchanged. Expression of *ppara* and *carnitine palmitoyltransferase 1a* were unchanged.

Additionally, we measured the expression of *srebp1c*, *diacylglycerol acyltransferase 2*, a key acyl transferase, and *pgc1a*, a master regulator of mitochondrial biogenesis. All were unchanged. Finally, we measured the expression of adipokines that can improve insulin sensitivity, including white adipose derived *adiponectin*, *leptin*, and *fgf21*. The mRNA expression of all of these genes was not significantly changed.

### T2 treatment and hepatocellular stress

**Fig 4:**
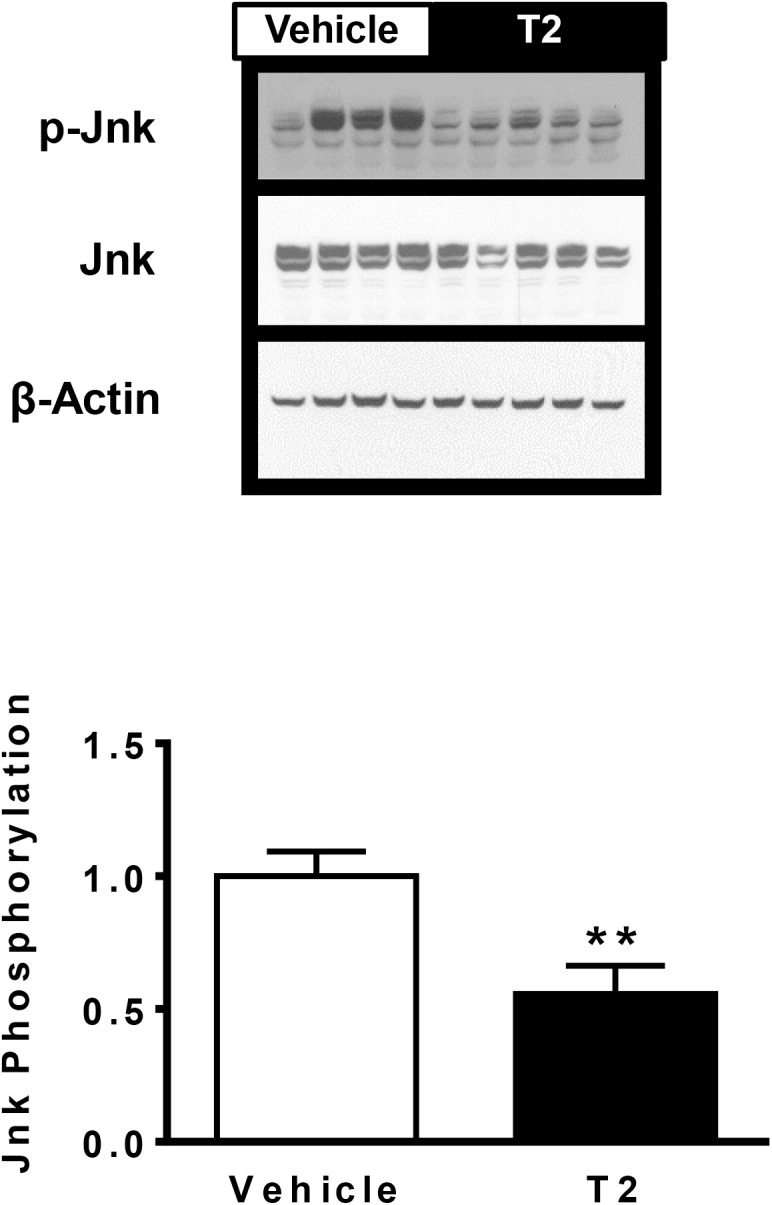
Hepatic c-Jun N-terminal kinase (JNK) phosphorylation assessed by Western blot. Representative immunoblots displayed. Data quantitated as ratio of phosphorylated JNK to total JNK abundance, normalized to vehicle treatment. (** P < 0.01; Vehicle treatment: *n* = 8; T2 treatment: *n* = 9).

Though T2 did not alter hepatic lipid content or hepatic glucose metabolism in response to insulin, there were changes in canonical insulin signaling pathways that merited further investigation. Multiple pathways may impact insulin signaling, including the unfolded protein response ("endoplasmic reticulum stress") and inflammatory cytokine signaling pathways [28]. There was no change in the expression of proteins that mediate ER stress, such as glucose regulated protein 78 (GRP78, or "BiP") or calnexin (S1 Fig) with T2 treatment. Drosophila tribbles homologue 3/ Tribbles pseudokinase 3 (Trb3) can directly connect the unfolded protein response to inhibition of Akt phosphorylation in several tissue types [31–33], but no change was seen in the expression of this gene (Table 4, *P* = 0.6). Inflammosome activation as assessed by procaspase 1 cleavage was not significantly reduced by T2 treatment (procaspase 1 cleaveage decreased by 19%, *P* = 0.16, S2 Fig). Many hepatic inflammatory pathways and the unfolded protein response converge on c-Jun N-terminal kinase (JNK) phosphorylation. There was a 44% reduction in JNK phosphorylation in T2 treated rats, as compared with vehicle-treated animals (Fig 4, *P* < 0.01). JNK activation is thought to impact energy balance by indirectly repressing peroxisome proliferator-activated receptor α (PPARα) activity through reductions in the expression of PPARα corepressors NCoR and Nrip, leading to decreased PPARα-regulated FGF21 expression, driving alterations in lipid and ketone body metabolism [34]. However, we observed no difference in expression of *NCoR* or *Nrip* (Table 4), and *fgf21* trended lower (hepatic *fgf21* expression decreased by 47%, *P* = 0.06, Table 4), arguing against PPARα activation. Thus, while the reduced activation of JNK is intriguing, this did not impart meaningful changes in putative downstream pathways nor did it ultimately improve hepatic insulin sensitivity.

## Discussion

Diiodothyronine is an intriguing pharmacologic candidate for the treatment of NAFLD. Though it is naturally occurring, and considered a physiologically inert product of iodothyronine metabolism, multiple studies demonstrate its lipid lowering effects at pharmacological doses [19–21]. Reduction in hepatic lipid content, specifically diacyglycerol content, could result in decreased activation of PKCε and improve fat-induced hepatic insulin resistance [28]. However, the therapeutic potential of T2 requires further vetting with both detailed mechanistic studies and whole-animal physiology studies.

Many gaps exist in our understanding of the mechanism whereby T2 may produce physiological actions. For example, the relative importance of TR-dependent actions of T2 as compared with the TR-independent actions of T2 is unclear. And, if the TR-independent actions of T2 are physiologically important, the existence of a discrete T2 sensitive receptor that mediates these actions is unknown. In vivo and in vitro studies suggest that T2 may be 100 [17] to 100,000 [35] less potent at activating nuclear thyroid hormone receptors than T3. Cholesterol reduction is seen at 12.5 mg/kg-d [13], a very high dose which likely activates TR. Even at 750 μg/kg-d T2 suppresses TSH and promotes cardiomegaly in rodents [23]. The most commonly studied dose in rodents is 250 μg/kg-d. Reports suggest that this dose does not activate TR [36, 37], and it may exert pharmacological effects by promoting deacetylation and activation of SIRT1 [18].

Contrary to prior reports, T2 treatment at 250 μg/kg-d failed to alter adiposity or hepatic steatosis either after a short-term (10 days) or long-term (4 weeks) course of treatment. Consistent with these findings, there were no changes in glucose metabolism or insulin action. Basal EGP and insulin-stimulated whole-body glucose disposal were matched between the vehicle and T2 treated groups. Moreover, the ability of insulin to suppress EGP was unchanged. The absence of an effect on insulin action was consistent with the findings that T2 did not reduce hepatic lipid content or PKCε activation. Interestingly, T2 treatment did augment insulin stimulated phosphorylation of IRK and Akt2. However, this effect on IRK and Akt2 phosphorylation may be primarily attributable to changes in basal phosphorylation state of these proteins. Additionally, this dynamic change in protein phosphorylation of IRK and Akt2 did not extend to FOXO1, a key downstream target of insulin signaling.

We explored other potential cellular responses to T2 treatment. There was no change in the expression of proteins associated with the unfolded protein response, and there was no significant change in procaspase 1 cleavage, both of which are components of pathways that impact insulin signaling. In contrast, we found a reduction in JNK phosphorylation after T2 treatment. JNK activation has been associated with impaired insulin signaling and altered energy metabolism. Though the reduction in JNK phosphorylation is consistent with the subtle increases in insulin stimulated Akt phosphorylation, this did not impact hepatic insulin action (e.g. suppression of EGP) to a meaningful degree. JNK activation has been associated with decreased lipid oxidation attributed to a decreased expression of *ppara* and *fgf21* (a PPARα target) expression. However, hepatic lipid content was not decreased, and *fgf21* expression was not increased, by T2 treatment. Thus, the changes in JNK phosphorylation, while intriguing, appear isolated from physiologically meaningful actions Prior reports documented numerous improvements with T2 treatment which were not evident in the present study. One notable difference between this study and the previous reports is in the diet: a unsaturated fat-predominant plant oil based diet (this study) as compared with a saturated fat-predominant animal fat based diet (prior studies). The mechanism proposed for hepatic T2 action is dependent on SIRT1 activation [18], and there is precedent for differential SIRT1 activation in animals fed diets that are predominant in saturated vs. unsaturated fat. A saturated fat diet was seen to be protective from the development of fatty liver in ethanol fed mice through SIRT1 mediated deacetylation of SREBP-1 and FOXO1 [38, 39]. The protective effect of T2 against the development of NAFLD may be blunted by a diet predominantly composed of unsaturated fat. Another potential explanation may be strain-specific differences in response to pharmacologic T2. The prior studies were performed in Wistar rats, while this study was performed with Sprague Dawley rats, and differences in both lipoprotein metabolism and endocrine function have been noted between these two strains [40, 41]. Thus, the effects of T2 on hepatic lipid metabolism may be influenced by specific diet conditions or limited to certain preclinical models.

In the setting of the rising morbidity of obesity, insulin resistance, and NAFLD, thyroid hormone analogues and metabolites present a promising avenue for the discovery of new, much needed pharmacologic agents. The goal for this class is to subtly alter hepatic lipid metabolism from efficient energy storage to uncoupled energy expenditure, while minimizing side effects. While some studies do suggest that T2 has clinical utility, our data demonstrate that T2 does not reduce hepatic steatosis or improve hepatic insulin sensitivity in in safflower-oil fed male SD rats. While the present studies do not support the clinical utility of T2 per se, they do demonstrate that T2 is not an inert metabolite and does exert some effects. Additional studies are required to further delineate the cellular mechanisms whereby T2 acts and the specific contexts in which it may be efficacious. T2 may act at novel targets which could be leveraged for therapeutic efficacy by more selective and potent compounds.

## Supporting Information

S1 Dataset (XLSX)

S2 Dataset (PDF)

S1 Fig Hepatic glucose regulated protein 78 (BiP) and calnexin protein abundance. Assessed by Western blot. Representative immunoblots displayed. Data quantitated as ratio with β-actin content, and normalized to vehicle treatment. (Vehicle treatment: *n* = 8; T2 treatment: *n* = 9)
(PDF)

S2 Fig Hepatic procaspase 1 cleavage. Assessed by Western blot. Representative immunoblots displayed. Different exposure times were required to quantitate caspase 1 p10 and procaspase 1 abundance; caspase 1 p10: procaspase 1 ratio normalized to vehicle = 1. (Vehicle treatment: *n* = 8; T2 treatment: *n* = 9).
(PDF)
