# Supplementary material for: 3,5 Diiodo-L-Thyronine (T2) Does Not Prevent Hepatic Steatosis or Insulin Resistance in Fat-Fed Sprague Dawley Rats
Source: PLoS One. 2015 Oct 20;10(10):e0140837. doi: 10.1371/journal.pone.0140837 (PMC4618341; doi:10.1371/journal.pone.0140837)

Total IRK

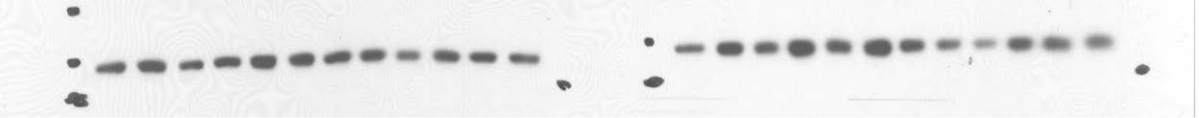

Phospho IRK

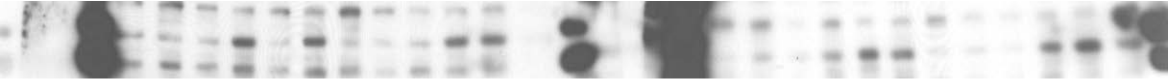

Total FOXO

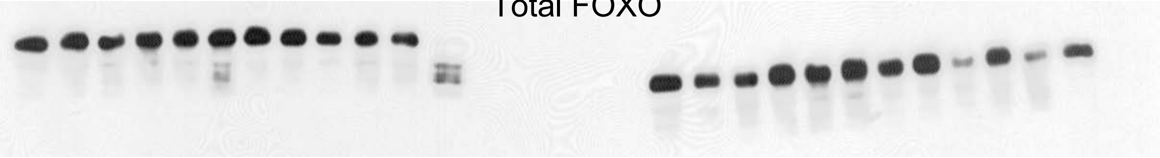

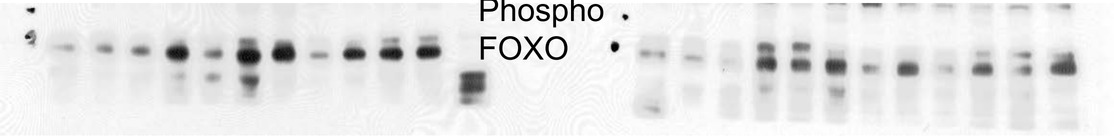
Phospho
FOXO

PKCe cytoplasm

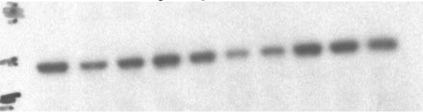

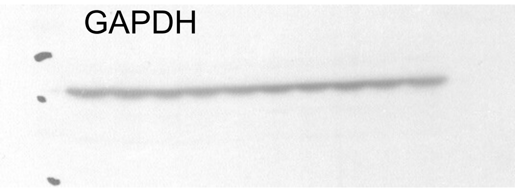
GAPDH

PKCe Plasma Membrane

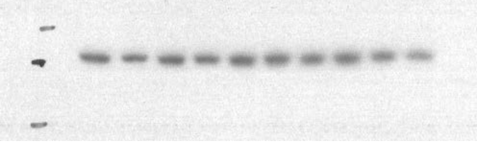

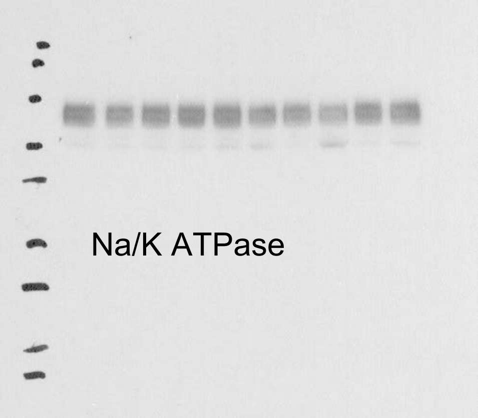
Na/K ATPase

Total Akt2

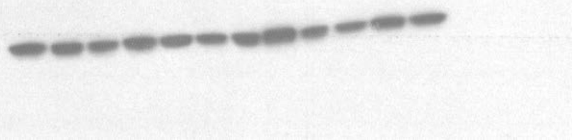

PhosphoAkt2

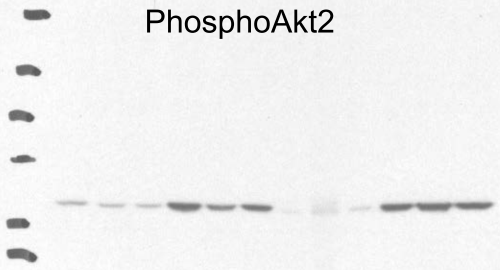

Total JNK

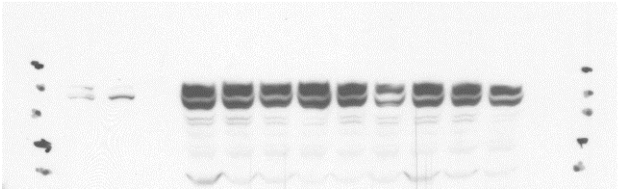

Phospho-JNK

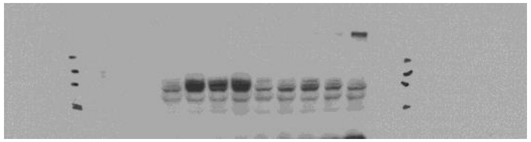

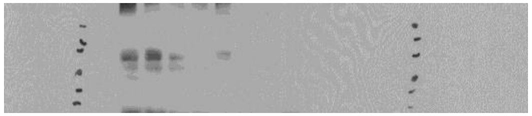

BiP

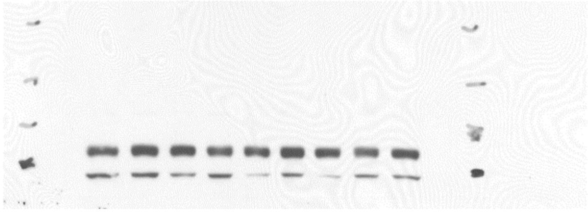

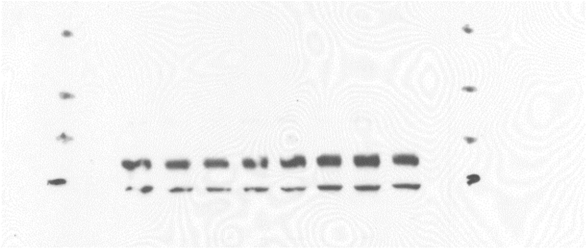

Calnexin

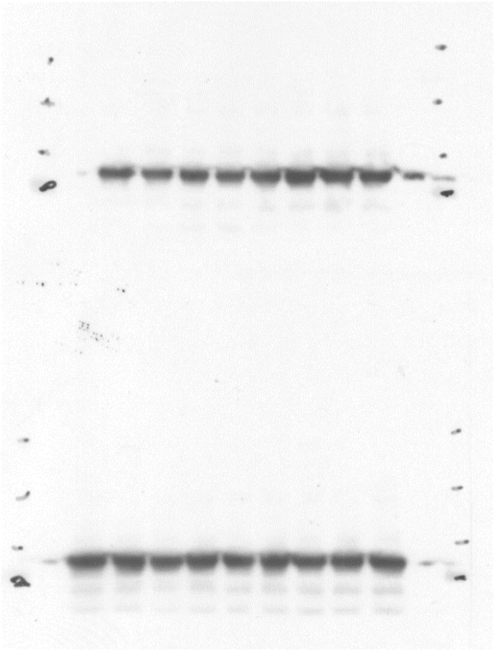

Procaspase 1

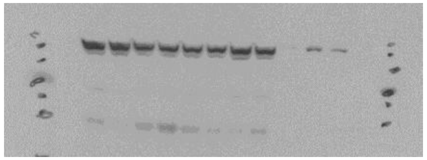

Caspase 1 p10

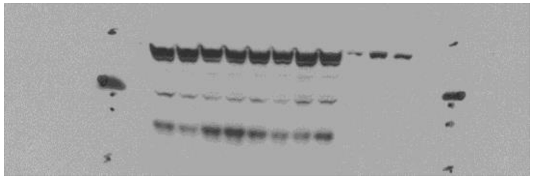

β-Actin

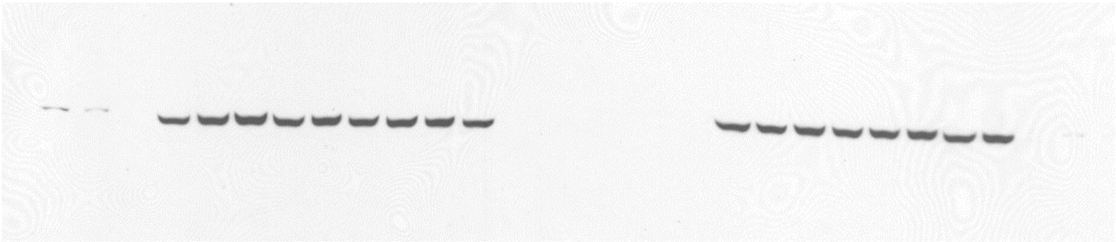

Supplement: S2 Dataset — (PDF) [file pone.0140837.s002.pdf]
