# Supplementary figures and images for: 3,5 Diiodo-L-Thyronine (T2) Does Not Prevent Hepatic Steatosis or Insulin Resistance in Fat-Fed Sprague Dawley Rats
Source: PLoS One. 2015 Oct 20;10(10):e0140837. doi: 10.1371/journal.pone.0140837 (PMC4618341; doi:10.1371/journal.pone.0140837)

Figure S1

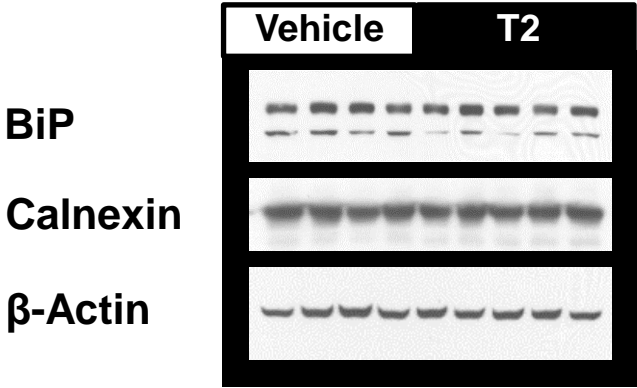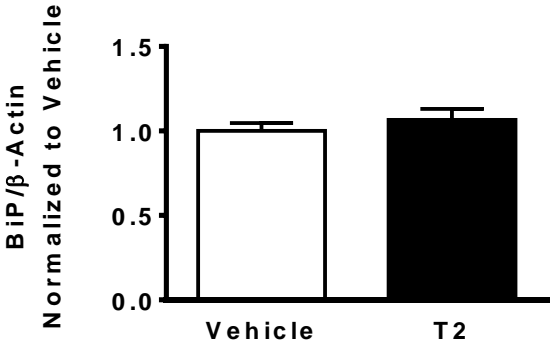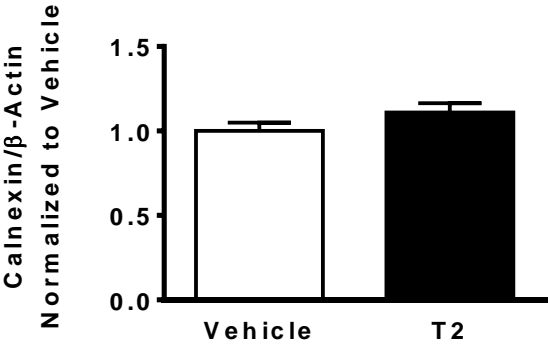

Supplement: S1 Fig — Assessed by Western blot. Representative immunoblots displayed. Data quantitated as ratio with β-actin content, and normalized to vehicle treatment. (Vehicle treatment: n = 8; T2 treatment: n = 9) (PDF) [file pone.0140837.s003.pdf]

**Figure S2**

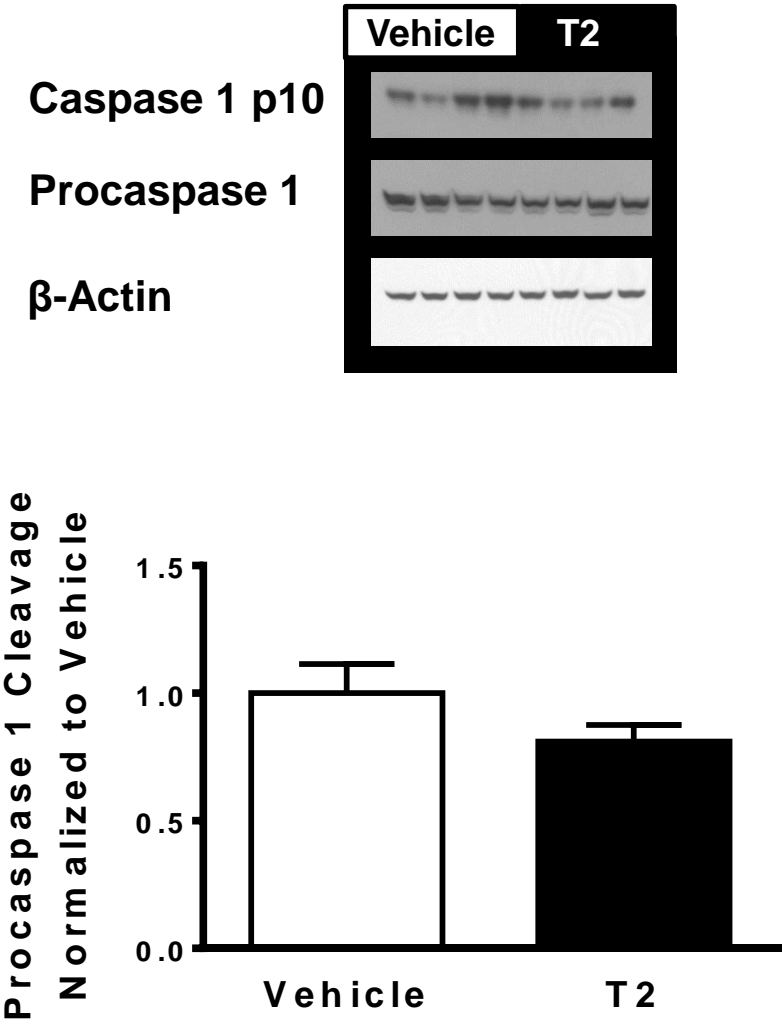

Supplement: S2 Fig — Assessed by Western blot. Representative immunoblots displayed. Different exposure times were required to quantitate caspase 1 p10 and procaspase 1 abundance; caspase 1 p10: procaspase 1 ratio normalized to vehicle = 1. (Vehicle treatment: n = 8; T2 treatment: n = 9). (PDF) [file pone.0140837.s004.pdf]
